# The Influence of Previous Experience on Virtual Reality Adoption in Medical Rehabilitation and Overcoming Knowledge Gaps Among Health Care Professionals: Qualitative Interview Study

**DOI:** 10.2196/62649

**Published:** 2025-04-30

**Authors:** Melina Schreiter, Jasmin Hennrich, Anna Lina Wolf, Torsten Eymann

**Affiliations:** 1 FIM Research Center for Information Management Bayreuth Germany; 2 Branch Business & Information Systems Engineering Fraunhofer FIT Bayreuth Germany; 3 Chair of Information Systems Management and Digital Society University of Bayreuth Bayreuth Germany

**Keywords:** virtual reality, technology adoption, medical rehabilitation, qualitative content analysis, adoption decision process, health care professionals, previous experience

## Abstract

**Background:**

Virtual reality (VR) technologies in health care, particularly in medical rehabilitation, have demonstrated effectiveness by enabling patient remobilization in virtual environments, offering real-time feedback, enhancing physical function and quality of life, and allowing patients to exercise autonomously. Nevertheless, VR technologies are facing slow adoption in routine rehabilitative practice due to health care professionals’ concerns regarding data security, lack of time, or perceived cost.

**Objective:**

This study aimed to explore how previous experience with VR technologies influences health care professionals’ decisions to adopt or reject these technologies in medical rehabilitation.

**Methods:**

We conducted 23 semistructured interviews with health care professionals from different rehabilitative fields in Germany, whom we grouped into VR-experienced “innovators” and nonexperienced “laggards” according to their level of innovativeness. When analyzing the interviews, we applied qualitative content analysis techniques and derived 56 preliminary categories from the transcripts.

**Results:**

We merged the preliminary categories into 26 adoption and rejection factors, which were grouped under the 4 overarching categories of the diffusion of innovation theory by Rogers. In addition to the pure identification of context-specific influencing factors, we were able to identify differences between these factors concerning the two different adopter groups. VR-experienced innovators exhibited key characteristics such as openness to new technologies, solution-oriented thinking, and opinion leadership, whereas nonexperienced laggards focused on barriers and relied on top-down knowledge transfer. Despite these differences, both groups agreed on the factors that promote the adoption of VR technologies. Our results indicate that addressing the unique needs of both groups is crucial for wider VR acceptance in health care.

**Conclusions:**

This study demonstrates the importance of distinguishing between VR-experienced and nonexperienced health care professionals, providing actionable insights for developing adopter-specific communication strategies to overcome barriers and foster broader diffusion of VR technologies in the health care sector.

## Introduction

### Overview

Today’s advancements in technology are reshaping how individuals engage with their physical environment. Among these innovations, virtual reality (VR) has emerged as a transformative tool, enabling users to visualize and engage with a simulated environment using advanced computing techniques [1]. In recent years, the health care sector has increasingly recognized the transformative potential of VR, as evidenced by the growing volume of research in this field [2,3]. These technologies facilitate immersive training for health care professionals, virtual consultations for patients, and innovative therapeutic approaches. Most studies focus on evaluating the effectiveness of VR-based treatments in various areas of health care, such as psychiatry, surgery, teaching and training, telemedicine, and medical rehabilitation [4,5]. VR technologies can be particularly suitable in rehabilitation after surgery, allowing patients to begin remobilization in a virtual world [6]. Recent studies have underscored the positive impact of VR technologies on physical function and overall quality of life in patients with diverse medical conditions [7-9]. VR technologies have shown to be more effective than traditional rehabilitation programs in improving balance and walking tasks, as demonstrated in a study by Pazzaglia et al [9] focusing on Parkinson disease rehabilitation. VR-based medical rehabilitation provides real-time feedback on movement performance, which can positively influence both motivation and adherence [10]. Moreover, greater autonomy is seen as an added value of the technology as it allows patients to perform exercises without close supervision by a therapist [11].

Although the potential of VR technologies in medical rehabilitation is well known and researched, the clinical adoption of these technologies remains low [12,13]. To comprehend the reasons underlying the limited integration of VR technologies, it is imperative to delve into the perspectives of potential users [13,14]. In the clinical setting, barriers to widespread VR implementation span financial, environmental, technical, and attitudinal domains [12,15]. While therapists have often expressed positive interest in VR technology [16,17], common barriers to adopting VR as a therapy tool that have been cited include perceived cost [17-19], limited access to resources [18], time barriers [17,19], and lack of space or infrastructure [17,18]. Furthermore, among others, practical hurdles can be attributed to the negative effects of VR, including motion sickness, which limits the interaction with VR technology [20], and ocular system overload, which can lead to visual discomfort and fatigue [21]. Users and potential users also express challenges in introducing VR technologies, such as privacy and security, particularly regarding the storage and use of sensitive patient data [22], as well as uncertainties about the suitability of VR for specific patient populations, such as older adults [23].

Existing studies regarding the adoption of VR technologies in medical rehabilitation focus on a specific clinical condition (eg, stroke [16,19]), a specific medical rehabilitation area (eg, communication rehabilitation [17,18]), a specific patient group (eg, veterans [24]), or a specific group of health care professionals (eg, speech-language pathologists [17]). A comprehensive understanding of health care professionals’ perspective and influencing factors on the adoption of VR technologies in medical rehabilitation is missing. In addition, the existing studies predominantly base their findings on statements about the hypothetical use of VR technologies in medical practice as most of the study participants have not yet used VR technology in a professional context [18]. Thus, research is needed to comprehensively investigate the factors that influence the adoption of VR technology by health care professionals, not only among those without previous experience but especially by comparing them with individuals who have previous experience. This aligns with the research call by Bryant et al [18] to move beyond the hypothetical use of VR technologies. This investigation is paramount for discerning the determinants that have prompted VR-experienced health care professionals (“innovators”) to embrace VR technologies in contrast to health care professionals without VR experience (“laggards”), who may exhibit resistance to digital innovations. Following this call for research, this study addressed the following research question: How does previous experience with VR technologies influence health care professionals’ decisions to adopt or reject these technologies in medical rehabilitation?

We used the diffusion of innovation theory by Rogers [25] as the theoretical lens for answering this research question. This theory, widely used to study innovation adoption and diffusion, emphasizes categories of adopters, such as innovators and laggards; the adoption decision process; and the role of communication, making it particularly relevant for this study. It has been successfully applied in health care to analyze the adoption of digital technologies such as telemedicine and electronic health records [26]. Using this framework, we structured the analysis of 23 semistructured interviews with health care professionals both with and without VR experience. This theory guided us in identifying key factors influencing adoption decisions, including perceived innovation attributes (eg, compatibility, complexity, and relative advantage), individual characteristics, and communication dynamics between VR-experienced and nonexperienced professionals. This approach provided nuanced insights into how differences in experience influence barriers to and facilitators of adoption.

### VR in Medical Rehabilitation

Medical rehabilitation, which improves physical and mental functioning to reduce disability, is a core component of high-value care [27]. According to the World Health Organization [28], approximately 2.4 billion individuals worldwide live with conditions requiring medical rehabilitation. This number is expected to rise due to the demographic shift toward an older population with higher life expectancy [29]. Medical rehabilitation requires continuous monitoring, individualized treatment plans, and adaptations to the patient’s progress, and thus, it is very time-consuming and costly [30]. In light of the deteriorating conditions in the health care system, characterized by a rising demand for medical rehabilitation services coupled with a dwindling supply due to a shortage of health care professionals [31], there is a pressing need to explore innovative solutions.

The application of VR technologies in medical rehabilitation represents a promising innovative approach that enables spatially and temporally independent treatment of patients while reducing the workload of health care professionals [32]. The capability to simulate real-life situations visually and interactively can help improve the recovery of functions and abilities of patients [33]. VR-based medical rehabilitation shows potential in supporting patients with spinal cord injuries, brain injuries, and other conditions and can provide benefits in managing symptoms or improving quality of life for individuals with Alzheimer disease [34]. In spinal cord injury rehabilitation, VR technologies have been investigated as a tool for gait training. Studies have shown that patients who received VR-based gait training significantly improved motor function and skills, balance, and aerobic function [35]. VR technologies allow users to immerse themselves in an artificial, computer-generated environment and experience it interactively [36]. The user usually wears a headset that offers a 360° view of the virtual world and can control the environment with various input devices, such as hand controllers or motion sensors [37]. VR provides the user with a completely virtual environment separate from the physical space, ranging from low immersion to full immersion [38].

Despite VR technologies’ potential for medical rehabilitation, several adverse effects of exposure to VR environments have been well documented and may cause issues. Commonly cited are, for example, motion sickness [20,39], ocular system overload [21], decreased control of limbs and posture [40], decreased sense of presence [41], and the development of negative emotional reactions [42]. Despite significant technological advancements in recent years, adverse effects such as motion sickness (or cybersickness as a specific form) remain a challenge for some users as individual susceptibility and VR content design continue to influence the experience. These factors contribute to the ongoing hesitation among potential users regarding the adoption of VR technologies [43].

### Users’ Perception in VR Diffusion

Understanding the user’s perception of a technology is critical for its diffusion, adoption, and acceptance, which are key elements in the implementation of innovations [44-46]. While the technology acceptance model [47] and the Unified Theory of Acceptance and Use of Technology [48] focus primarily on the individual user’s intention regarding technology use, the diffusion of innovation theory extends the consideration to the diffusion of the innovation in a social system [25]. Thus, the adoption decision process is seen as a micro viewpoint on change, whereas diffusion is a macro perspective that describes how innovation spreads throughout a population [25]. According to Rogers [25], the adoption decision process is embedded in the diffusion process, implying that, if a critical mass of individuals adopts an innovation, it leads to the innovation’s diffusion to the broader population.

**Figure 1:**
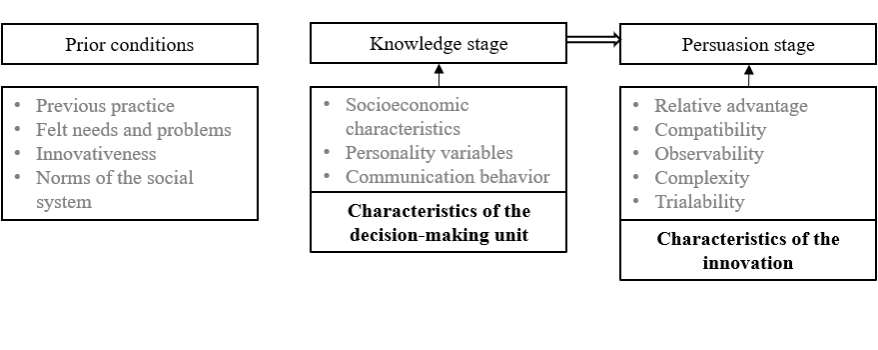
Prior conditions, knowledge stage, and persuasion stage within the adoption decision process (Rogers [25]).

To understand how individuals perceive innovation, we focus on the micro level, specifically examining prior conditions, knowledge, and the persuasion stage within the adoption decision process (Figure 1 [25]). Prior conditions include *previous practice*, *felt needs and problems*, *innovativeness*, and *the norms of the social system*. The first stage is about acquiring *knowledge* about the innovation, which Rogers [25] divides into awareness knowledge (knowing an innovation exists), how-to knowledge (knowing how to use an innovation properly), and principle knowledge (knowing why an innovation works). This knowledge is determined by the individual’s *socioeconomic characteristics*, *personality variables*, and *communication behavior*. In stage 2, *persuasion*, a positive or negative attitude regarding the innovation, is created, determined by 5 key characteristics of innovation: *relative advantages*, *trialability*, *compatibility*, *complexity*, and *observability*. *Relative advantage* refers to the perceived benefits of an innovation. *Trialability* refers to the ability to test an innovation before committing to it. *Compatibility* refers to how well the innovation integrates with potential adopters’ values and needs. *Complexity* measures how difficult an innovation is to understand, whereas *observability* is the visibility of the benefits of an innovation to potential adopters.

To understand the diffusion of innovations from a macro perspective, it is essential to consider the diffusion within a social system comprising different adopter groups [25]. Rogers [25] distinguishes between categories of adopters: innovators, early adopters, early majority, late majority, and laggards. These groups are based on their varying levels of motivation and willingness to adopt new technologies. Innovators are prepared to experience new ideas and have complex technical knowledge, whereas early adopters are more limited by the boundaries of the social system [25]. The attitudes of early adopters toward innovation are more important as role models. Rogers [25] assumed that, although the early majority interact well with other members of the social system, they do not have the leadership role that the early adopters have. Similarly to the early majority, the late majority comprises members of the social system who wait until most of their peers adopt the innovation. Although they are skeptical about the innovation and its outcomes, economic necessity and peer pressure may persuade them to adopt the innovation. As Rogers [25] found, the laggards have a traditional view and are more skeptical of innovation and change.

## Methods

A qualitative research approach is suitable for understanding and investigating health care professionals’ adoption or nonadoption in medical rehabilitation, going beyond simplified factors of existing adoption theories [49,50]. Using interviews, we aimed to ascertain the prevalent experiences, viewpoints, and emotions of two groups of health care professionals regarding VR technologies in medical rehabilitation.

### Interview Guideline

The interview guideline was developed using the 4-phase framework by Castillo-Montoya [51] for refining interview protocols. In the first phase, we mapped the interview questions onto the research question to ensure alignment. Following this, we revised our interview guideline to include practical terms that correspond to the interviewees’ terminology (eg, “use” instead of “adoption”; phase 2). In phase 3, we reflectively discussed and revised the first version of the interview guideline and created 2 versions (one for participants with and one for participants without VR experience). The people with VR experience were asked about their experiences, whereas a specific use case was created for people without VR experience, such as the following: “Imagine you are using VR...” During phase 4, the interview guidelines underwent pretesting with a prospective physician and a physical therapist. The final interview guidelines structured the interviews as follows. At the beginning of each interview, we gave a short overview of the research team and the aim of the study before requesting the interviewees to introduce themselves and their professional backgrounds. After establishing a comfortable atmosphere for discussion, we questioned the interviewees about VR technologies in their field of medical rehabilitation. We used open-ended questions to avoid biasing respondents in any direction and preserve the study’s exploratory nature [51]. The questions were similar to the following: “When you think of VR technologies, what are the first thoughts that come to your mind? and What is your take on the use of VR technologies in rehabilitation?” With participants’ consent, the interviews were audio recorded, transcribed, and analyzed using MAXQDA (VERBI GmbH). In total, we conducted 23 interviews via videoconference.

### Data Analysis

For the analysis of the interview transcripts, we followed the recommendations by Mayring [52] for mixed inductive and deductive category building using qualitative content analysis (QCA). We started with the inductive, data-driven development of categories and set 2 requirements for these categories. First, based on the research question, the categories should include factors that affect the adoption decision process of VR technologies in medical rehabilitation. The second requirement concerned the abstraction level of the concepts [52]. To achieve a certain degree of transferability of the results, the categories aimed to include content that was not unique to the interview participant but was also transferable to other individuals [53]. After formulating these requirements, the interview transcripts were independently analyzed by 2 researchers, who developed inductive categories. We created 56 preliminary inductive categories and recorded relevant information in memos. To place the identified categories in an appropriate context, we additionally coded statements from the interviewees regarding their understanding and experience of VR technologies and their perceived prevalence and future importance in medical rehabilitation. Mayring [52] recommends a revision after analyzing 10% to 50% of the data. After reviewing 38% of the material, the authors discussed the memos and categories, merged analogous categories, and came to a resolution regarding conflicting categories. During this process, we drew connections to the adoption decision process of the diffusion of innovation theory. Thus, the diffusion of innovation theory helped guide and structure the deductive coding phase, allowing us to contextualize our findings within a broader theoretical framework. For example, the inductive category *Obstructive properties of the technology* was mapped to the diffusion of innovation category *Complexity*. This allowed us to refine the concept of complexity further, breaking it down into 2 factors—*Complexity of VR hardware* and *Complexity of VR software*—that were particularly relevant to medical rehabilitation. Subsequently, we applied deductive codes to all the interview transcripts. Finally, 26 factors determining VR adoption or rejection in medical rehabilitation were identified and assigned to the 4 overarching categories of the diffusion of innovation theory by Rogers [25].

### Ethical Considerations

The Ethics Committee of the University of Bayreuth approved our research proposal (approval number 25-021) and checked if it was compliant with the General Data Protection Regulation. Before the interviews, all participants received a declaration of consent and information clearly stating that recordings would be used exclusively for research purposes and that all personal data would be pseudonymized and treated with strict confidentiality. Unique pseudonyms were used instead of names, and the data were securely encrypted. Participation in the interviews was completely voluntary without any compensation, and participants could withdraw at any time without any negative consequences.

## Results

### Descriptive Results and Study Population

We conducted expert interviews in Germany from February 2023 to April 2023. To ensure diverse perspectives, medical rehabilitation experts of different experiences with VR technologies, professional backgrounds, sex, and ages were included. The interview participants were selected according to the principle of convenience sampling. Finally, we interviewed 23 participants out of 36 contacted health care professionals, comprising physiotherapists (n=8, 35%), physicians (n=4, 17%), sports therapists (n=3, 13%), occupational therapists (n=2, 9%), nurses (n=2, 9%), speech and language therapists (n=1, 4%), osteopaths (n=1, 4%), psychologists (n=1, 4%), and medical-technical functional diagnosticians (n=1, 4%). The interviews lasted approximately 30 (SD 6.69) minutes on average, and a total of 627 interview minutes were included in the data analysis. The characteristics of the interviewees and their VR experience are listed in Table 1.

**Table 1:** Characteristics of the interviewees (N=23).

ID	Sex	Age (y)	Type of medical professional	VR^a^ experience
PT1	Male	25	Physiotherapist	None
PT2	Male	34	Physiotherapist	None (private)^b^
PT3	Male	30	Physiotherapist	Yes (professional)^c^
PT4	Female	26	Physiotherapist	None
PT5	Female	50	Physiotherapist	None
PT6	Male	25	Physiotherapist	None
PT7	Female	26	Physiotherapist	Yes (professional)
PT8	Male	36	Physiotherapist	Yes (professional)
D1	Male	33	Physician	None
D2	Female	27	Physician	None
D3	Male	63	Physician	Yes (professional)
D4	Male	45	Physician	None
ST1	Male	33	Sports therapist	None
ST2	Female	30	Sports therapist	Yes (professional)
ST3	Male	36	Sports therapist	Yes (professional)
OT1	Female	37	Occupational therapist	Yes (professional)
OT2	Female	32	Occupational therapist	Yes (professional)
N1	Male	46	Nurse	Yes (professional)
N2	Female	44	Nurse	Yes (professional)
LT1	Female	36	Speech and language therapist	None
O1	Male	58	Osteopath	Yes (professional)
P1	Female	32	Psychologist	Yes (professional)
MTF1	Female	30	Medical-technical functional diagnostician	None (private)

^a^VR: virtual reality.

^b^In the context of gaming.

^c^In the context of occupation.

### Coding Results

#### Overview

Aligned with our research goal, we explored factors influencing health care professionals’ adoption or nonadoption of VR technologies in medical rehabilitation. In this section, we present our findings of the QCA and support them with interview quotes. First, we introduce the interviewees’ experience and understanding of VR technologies. We then describe the 4 categories and 26 factors that determine VR adoption or rejection by health care professionals in medical rehabilitation. To deeply analyze the influencing factors on the adoption decision, the interview participants were categorized into two groups based on whether they had professional or no professional experience with VR technologies (Table 1). In the Previous Practice section, further reference is made to the subdivision.

All participants were aware of the existence of VR technologies and had some understanding of how these technologies work, although not all participants had concrete experience with the technology. Overall, 39% (9/23) of the participants had no previous experience with VR technologies. In contrast, 9% (2/23) of the participants had experience exclusively in a private context, and 52% (12/23) of the participants reported experience with VR technologies in their profession. Despite having a basic understanding of VR technologies, some interviewees could not imagine what VR technologies meant in medical rehabilitation. For example, one interviewee commented the following:

Yes. So, as I said, I cannot imagine anything about how the whole thing [VR technologies] is supposed to work...

PT4; no professional experience with VR

Furthermore, another participant stated the following:

I cannot imagine how you could do something rehabilitative for the patient [with VR technologies].

PT5; no professional experience with VR

In addition to this lack of understanding, health care professionals sometimes misunderstood VR technologies in medical rehabilitation because the “difference between therapy and game” (OT2; professional experience with VR) was unclear. All participants considered the prevalence of VR technologies in medical rehabilitation in Germany to be low. One interviewee said the following:

I would say it [VR technologies’ prevalence] is very low. Are there any [VR technologies] at all? Well, some pioneers are probably already working with it [VR technologies]. But that is probably really the minority...

MTF1; no professional experience with VR

In contrast, VR technologies are already being used more frequently in other countries, such as Japan (P1; professional experience with VR). In general, most participants were in favor of the future relevance of VR technologies in medical rehabilitation even though they assumed that the diffusion of VR technologies would take a while (MTF1; no professional experience with VR).

#### Prior Conditions

##### Overview

The adoption decision process of the diffusion of innovation theory is determined by the *prior conditions* and includes categories such as previous VR practices or norms of the social system. Table 2 provides an overview of the factors in medical rehabilitation identified through QCA, along with the number of text segments coded for each factor per experience group. The core message from the interviewees emphasized that previous experiences with VR technologies are crucial in shaping health care professionals’ willingness to adopt these technologies in medical rehabilitation.

**Table 2:** Results of the prior conditions.

Prior conditions and factors in medical rehabilitation		Number of coded text segments
**Previous practice**		
	No previous VR^a^ experience	Professional experience: 0 No experience: 11
	Previous VR experience	Professional experience: 12 No experience: 0
**Norms of the social system**		
	Patient perspective	Professional experience: 45 No experience: 47
	Exchange in the team	Professional experience: 6 No experience: 3
	External stakeholder	Professional experience: 52 No experience: 39
**Felt needs and problems**		
	Health care market uncertainties	Professional experience: 9 No experience: 10
	IT infrastructure	Professional experience: 22 No experience: 9
**Innovativeness**		
	Health care professional engagement	Professional experience: 18 No experience: 16
	Low affinity with technology	Professional experience: 15 No experience: 12

^a^VR: virtual reality.

##### Previous Practice

We used this factor to categorize the interview participants according to their previous VR experience. As shown in Table 2, of the 23 interviewees, 11 (48%) had no professional VR experience; however, of these 11 participants, 2 (18%) had previous private (gaming) experiences with VR technologies. Interviewees who had already been exposed to VR technologies in a private context recognized the potential of these technologies in their profession. For instance, one interviewee commented the following:

I put on [VR] glasses in a museum...and [then very quickly] this therapeutic idea was born in me.

OT2; professional experience with VR

Another participant stated the following:

[I was once in] an exhibition of, I think it was Daimler...and I found that very fascinating.... [Shortly after that] I witnessed a startup, and that is when I got a real insight into what potential VR technologies have in rehabilitation.

D3; professional experience with VR

On the other hand, interviewees with *no previous VR experience* tended to express themselves in a rather anxious and reserved manner:

...I will be honest. I have a lot of respect for it [VR technologies]. But I have not had that experience with it [VR technologies], so I could say that it catches me or that I cannot do anything with it.

PT4; no professional experience with VR

The other interview group, comprising 52% (12/23) of the participants, had already gained VR experience in a professional context, ranging from at least once to daily.

##### Norms of the Social System

This category refers to 3 stakeholders within the health care professionals’ environment that influence their adoption decision: patients (92 coded segments); the team of the health care professionals (9 coded segments); and the outer setting, such as health insurance companies, legislation, and the organization that the health care professionals work in (91 coded segments). Regarding the *patients’ perspective*, one interviewee stated the following:

Maybe It [VR technologies] could fail in the sense that patients do not accept it, that they just say that it does not do anything for me or that confuses me, or I do not know how to do it right there.

D2; no professional experience with VR

Another interviewee considered it important to bring the technology closer to the patient:

...if you explain it [VR technologies] to them well and make it palatable...I do not think [patients] are then averse to it.

MTF1; no professional experience with VR

One occupational therapist asserted that patients listen to the therapist’s recommendation:

...I notice that so clearly. They [patients] are open to it. If I sit in front of them and say we are doing therapy today with VR technologies, and it can be an 80-year-old grandma, she does not say no...

OT2; professional experience with VR

Regarding the age of patients, VR users held a different view from that of non–VR users. One sports therapist said that she “would not see it [age] as a barrier, but rather as an opportunity” (ST2; professional experience with VR). Another interviewee could envision the use of VR technologies among certain age groups to a lesser extent:

...those who are not so technically skilled...so with over-60s, over-70s, over-80s.

T1; no professional experience with VR

Regarding the health care professionals’ *team*, one sports therapist noted that a new colleague with affinity for technology introduced VR glasses to the team and it “is only a matter of time [before] VR glasses are in our house too” (ST3; professional experience with VR). Some respondents indicated that they involved their team in the adoption decision:

I always present such newer things to the team [and] the decision is then also [made] in the team.

OT1; professional experience with VR

It emerged from the interviews that the *external stakeholder* is related to the adoption decision of health care professionals. VR-experienced health care professionals had already actively explored financing options, particularly through health insurance companies, and had also dealt with the legal aspects. Interviewees agreed that financial support from health insurers may be key to VR adoption. One interviewee commented the following:

Otherwise, we would have made the investment [in VR technologies] a long time ago.

ST2; professional experience with VR

In addition, health care professionals called for anchoring in therapeutic guidelines and recommendations, such as the S3 guidelines, to decide which VR technologies they should use in practice (ST2; professional experience with VR). The S3 guidelines are evidence-based clinical practice guidelines in Germany developed through a systematic process to ensure safe and effective interventions [54]. VR technologies must first be approved for medical purposes. One participant cited complexities related to the Medical Device Regulation to bring VR technologies to market as medical devices (P1; professional experience with VR). One respondent attributed responsibility to the employer (organization) when asked why she was not yet using VR technologies:

...[it] was also not yet required [from the clinic] to be done that way.

ST1; no professional experience with VR

Another participant hoped to see an increase in innovation in the organizations:

Who is sitting there in the rehabilitation clinics with an open mind?...and how open is the [CEO] really to innovation?

D3; professional experience with VR

The interviewees often attributed the decision-making power to the practice owner or management. Nevertheless, they saw themselves as responsible for recommending VR technologies to top management and convincing them of their potential benefits (ST1; no professional experience with VR).

##### Felt Needs and Problems

Health care professionals cited *market uncertainties* 19 times in the interviews as factors that influenced their adoption decisions independently of their VR experience. One physician saw VR technologies as a tool for the future to meet the requirements of the circumstances:

Well, in principle, it [VR technologies] will already play an important role because simply the demand for rehabilitation is growing, because the population is getting older...But there will also be fewer specialists. Then you must look for alternatives to somehow maintain the rehabilitation programs without worsening the outcome or just not achieving it...

D2; no professional experience with VR

The growing shortage of health care professionals makes it necessary to stand out from the competition in finding new employees. One participant was certain that VR technologies make this possible:

We wanted to remain competitive as a unique selling point...Of course, it is also special when I am looking for an employee and have VR glasses there.

PT7; professional experience with VR

Another factor influencing the adoption decision of VR technologies was the *IT infrastructure* in health care professional facilities, appearing 31 times in the interviews. One interviewee said that “a big barrier overall is implementing it [VR technologies] into existing structures because they have to be flexible and kind of make room” (D3; professional experience with VR). In particular, the “spatial requirements” (ST1; no professional experience with VR) would also have to be created. One participant stated that “[the] integration of VR technologies should result in an overall concept” (PT8; professional experience with VR).

##### Innovativeness

The *engagement* of the health care professionals in the adoption of technological innovations such as VR in clinical and therapeutical work was thematized in 34 interview quotes. The ability of health care professionals to innovate in the use of VR technologies was a crucial aspect as they “...also have to look for themselves what they feel comfortable with... [so that] self-interest develops” (ST3; professional experience with VR). Some interview participants saw the “lack of motivation...as the biggest barrier” (D3; professional experience with VR) to the adoption of VR technologies. One interviewee said the following:

So, I am open to that kind of stuff [VR technologies]. I also like to deal with such things [VR technologies]. I think it [VR technologies] are totally cool.

PT7; professional experience with VR

Another interviewee mentioned the following:

So, I do not see why I should use it [VR technologies] now.

PT6; no professional experience with VR

The *affinity for VR technologies* emerged 27 times as an influential factor in health care professionals’ adoption decisions. One interviewee cited his staff’s fears regarding their IT competencies during the VR adoption phase:

There was a lot of fear: Oh God, so much technology. I cannot do this anyway. It is just too technically demanding for me. I am already overwhelmed if I have to kind of reset my laptop at home or run a virus program on it...It took a while for them to accept that it is not that bad...

PT8; professional experience with VR

In addition, one speech and language therapist explained that her reluctance to use IT prevented her from adopting VR technologies:

I sometimes have a hard time with the latest innovations and with technology in general. That is why it takes me a little longer [and] when I can, I like to skip things like [VR technologies].

LT1; no professional experience with VR

#### Characteristics of Health Care Professionals

The characteristics of the decision-making unit are crucial when it comes to a person passing from the stage of knowledge to the stage of persuasion [25]. This aspect comprises 3 main categories within the diffusion of innovation theory: socioeconomic characteristics, personality variables, and communication behavior (Table 3). The interviewees expressed differing opinions on whether the high average age of health care professionals influences the adoption of VR technologies. In addition, health care professionals with VR experience often took on the role of opinion leaders, promoting VR through seminars and social media, thus influencing the adoption process.

**Table 3:** Results of the characteristics of health care professionals.

Characteristics of the decision-making unit	Factors in medical rehabilitation	Number of coded text segments
Socioeconomic characteristics	High average age	Professional experience: 0 No experience: 5
Personality variables	—^a^	—
Communication behavior	VR^b^-related interaction patterns	Professional experience: 11 No experience: 6

^a^Not applicable.

^b^VR: virtual reality.

Interviewees disagreed about whether *high average age* as a socioeconomic characteristic plays a role in adopting VR technologies. This factor was addressed a total of 11 times. One interviewee said the following:

...and my own experience is that older therapists [are] not so good with technology.

P1; professional experience with VR

An older participant explained the following:

These technical things, that is what my daughter [as a physiotherapist] does a lot, and [if it does not work right away] then I lack access, and I just leave it.

PT5; no professional experience with VR

In contrast, one interviewee held a diverging opinion:

There are definitely therapists, doctors, and colleagues in middle age or older who receive technology enthusiastically and also pass it on.

ST1; no professional experience with VR

The interview data did not allow for interpretation regarding the personality variables, which are reportedly challenging to identify [25]. The *VR-related interaction patterns*, which describe how individuals communicate with each other, particularly concerning the dissemination of technologies such as VR, were thematized in 17 interview quotes. Health care professionals with VR experience had gained access to VR through testing it in seminars and through social media. Furthermore, individuals from the group of health care professionals with VR experience expressed themselves as opinion leaders:

I scheduled an appointment today at the school...VR and neurofeedback will be one of the main points I introduce there.

OT2; professional experience with VR

#### Knowledge of Health Care Professionals

On the basis of the results of the interviews, and in accordance with Rogers [25], we categorized knowledge into 3 types: awareness knowledge, principle knowledge, and how-to knowledge. Table 4 provides an overview of the factors identified. The core opinion of the interviewees highlights that health care professionals’ limited awareness knowledge of VR technologies is a barrier to adoption in medical rehabilitation, with VR-experienced individuals emphasizing the need for increased education, training, and public awareness efforts.

**Table 4:** Results of the knowledge of health care professionals.

Characteristics of the decision-making unit	Factors in medical rehabilitation	Number of coded text segments
Awareness knowledge	Awareness knowledge	Professional experience: 8 No experience: 8
Principle knowledge	Principle knowledge	Professional experience: 4 No experience: 0
How-to knowledge	How-to knowledge	Professional experience: 22 No experience: 15

First, there is the *awareness knowledge* of the existence of an innovation, which was addressed 16 times in the interviews. Second, there is *principle knowledge* about how and why the innovation works, which was only mentioned a total of 4 times in the interviews and only among individuals who had experience with VR technologies. Third, knowledge enables the correct application of an innovation (*how-to knowledge*), which was discussed 37 times. In particular, interviewees with VR experience noted a limited level of awareness knowledge among health care professionals regarding the existence of VR technologies in medical rehabilitation. For instance, one interviewee mentioned the following:

My father [as a physiotherapist] also said...he would never have thought of getting something like that because he does not know it [VR technologies].

ST2; professional experience with VR

Another interviewee noted that “a lack of being informed about it [VR technologies]” can be considered one of the “biggest obstacles [to successful adoption]” (D3; professional experience with VR). This missing knowledge should be addressed by more “clearing-up work” (OT2; professional experience with VR) in schools, in education, and at therapy fairs, as well as an “intensified medium appearance...in the sense of television contributions” (ST2; professional experience with VR) or “rehabilitation magazines” (D3; professional experience with VR). Health care professionals with VR experience mentioned the appropriate use of innovation as an important adoption factor. To be able to use VR technologies properly therapeutically, “education” (OT2; professional experience with VR) and “training courses” (PT7 and OT1; professional experience with VR) were most relevant to VR-experienced interviewees. Marketing activities, such as those on social media, at trade shows, or other events, can help raise awareness of the benefits and applications of VR technologies. Conversely, respondents with no VR experience emphasized the need for leadership initiatives from senior individuals and rehabilitation institutions to promote the awareness and adoption of such technologies. They advocated for top-down VR promotion, hoping that leaders and rehabilitation institutions would actively drive these innovations forward and serve as role models.

#### Characteristics of VR Technologies

##### Overview

The adoption of an innovation is typically influenced by the perceived *characteristics of the innovation*, including relative advantage, complexity, trialability, observability, and compatibility [25]. Table 5 provides an overview of the arrangement of these factors in medical rehabilitation as well as their frequency of occurrence in our interviews. Interviewees emphasized the relative advantages of VR technologies in medical rehabilitation, highlighting improvements in patient outcomes, efficiency, and diversification. However, concerns about side effects, costs, complexity, data security, and compatibility with existing systems were raised, with those with VR experience viewing it more favorably in terms of practicality, whereas nonusers sought more opportunities for trial.

**Table 5:** Results of the characteristics of virtual reality (VR) technologies.

Characteristics of the innovation and factors in medical rehabilitation			Number of coded text segments
**Relative advantage**			
	Effectiveness	Professional experience: 84 No experience: 53	
	Efficiency	Professional experience: 37 No experience: 34	
	Diversification	Professional experience: 20 No experience: 39	
	Affordability	Professional experience: 25 No experience: 26	
	Data security	Professional experience: 4 No experience: 8	
**Complexity**			
	Complexity of VR hardware	Professional experience: 13 No experience: 9	
	Complexity of VR software	Professional experience: 24 No experience: 25	
**Trialability**			Professional experience: 7 No experience: 5
**Observability**			
	Lack of evidence-based studies	Professional experience: 9 No experience: 6	
	Measurability of results with VR technologies	Professional experience: 15 No experience: 5	
**Compatibility**			
	Compatibility with the existing values and needs of health care professionals	Professional experience: 27 No experience: 22	
	Compatibility with existing IT systems	Professional experience: 4 No experience: 5	

##### Relative Advantage

The relative advantage was found in a total of 330 text passages in the interviews, divided into effectiveness, with 137 (41.5%) coded text segments; efficiency, with 71 (21.5%) coded text segments; diversification, with 59 (17.9%) coded text segments; affordability, with 51 (15.5%) coded text segments; and data security, with 12 (3.6%) coded text segments.

*Effectiveness* was discussed more by health care professionals with VR experience compared to those without VR experience (84 coded text segments vs 53 coded text segments, respectively), but for almost all respondents, the effectiveness achieved with VR technologies was an important indicator for the adoption or rejection of the technology. For example, one interviewee said the following:

But if...the success that is achieved is not as great as if I were practicing with the patient in real-time. Then that would be again rather against the use of the VR technologies.

PT3; professional experience with VR

For respondents, a better outcome was conditioned by a “higher motivation of the patient” (ST3 [professional experience with VR] and D1 and ST1 [no professional experience with VR]), an “increased self-efficacy” (D3; professional experience with VR), an “increased compliance” (ST1; no professional experience with VR), the “distraction from the disease” (PT1; no professional experience with VR), and the “closeness to everyday life” (P1 and OT1; professional experience with VR). Furthermore, interviewees also questioned whether the side effects of VR technology use would have an impact on the effectiveness of the therapy, for example, “additional screen time,” the risk of “various children and adolescents becoming addicted,” and the fact that “body awareness is lost” (ST1; no professional experience with VR). Respondents who were already using VR technologies mentioned side effects such as “headaches” (OT2 and PT7; professional experience with VR), “eye pain” (PT7; professional experience with VR), “nausea” (OT2; professional experience with VR), “dizziness” (PT7; professional experience with VR), and the risk of “motion sickness” (OT1 and PT3; professional experience with VR). *Efficacy* encompassed statements about being able to work faster (eg, time efficiency), conserving resources (eg, group therapy), and operating more economically (eg, cost-benefit ratio) with VR technologies. This factor was addressed 37 times by health care professionals with VR experience and 34 times by health care professionals without VR experience. They differed in terms of perceived opportunities or concerns. On the one hand, individuals with no VR experience expressed concerns regarding training time and effort. The introductory phase of VR technologies “seems to initially involve more work” (LT1; no professional experience with VR). One interviewee stated the following:

The time investment required to engage with the technology is a tragic hurdle.

D2; no professional experience with VR

On the other hand, those with VR experience discussed a temporal relief in their work. They expressed a desire for “time savings” (PT3; professional experience with VR) resulting from “autonomous patient therapy from home without supervision” (ST1; professional experience with VR), the possibility of “group therapy” (PT7; professional experience with VR), and “automated documentation and measurement” (PT7; professional experience with VR). *Diversification* encompassed statements regarding portfolio expansion through the integration of VR technologies. Portfolio expansion entails the augmentation of conventional therapeutic interventions through technologies such as VR. This factor was more frequently addressed by individuals without VR experience, with 39 coded text segments, compared to those with VR experience, with 20 coded text segments. In terms of content, the interviewees agreed that the technology acts as a “support” (D4 and PT5; no professional experience with VR). One participant said the following:

It [VR technologies] makes me more competent as a therapist.

OT1; professional experience with VR

Nevertheless, she would never let “[VR technologies] stand alone as a base” (OT1; professional experience with VR). Furthermore, we understood diversification to mean individualized and patient-centered therapy, which respondents perceived as important. One interviewee described the following:

It [VR technologies] should be applicable to many patients, but still customizable. It should be for any age, for anybody constitution, or even for athletes, non-athletes, injuries, neurological, orthopedic...there should be enough programs that can then be used.

PT6; no professional experience with VR

Furthermore, a medical-technical functional diagnostician described the following:

...that it [VR technologies] should be personalized for the patient, that you can maybe create a familiar environment because it is always a more comfortable feeling for the patient than something foreign.

MTF1; no professional experience with VR

*Affordability* was addressed with comparable frequency among health care professionals both with and without VR experience, mentioned 25 and 26 times, respectively. However, there were distinctions in their perceptions of costs. While predominantly health care professionals who did not use VR technologies assessed the cost as still “very dauntingly high” (PT1; no professional experience with VR), one VR user who had already invested in the technology assessed the actual cost as not too high. In doing so, one interviewee described the investment in VR technologies as “one of the smallest investments [made] here in practice compared to other therapy methods” (OT2; professional experience with VR). In addition to pure acquisition costs, concerns were also raised about “development costs” (ST1 [no professional experience with VR] and PT3 [professional experience with VR]) and the potential cost of “spare parts” (ST1; no professional experience with VR). With the help of “leasing options” (ST2 and OT1; professional experience with VR), the cost risk of VR technologies could be mitigated. *Data security* was addressed by participants with VR experience 4 times, whereas those without VR experience discussed it twice as frequently. Overall, the interview participants held differing opinions on this matter. For example, one interviewee without VR experience said the following:

As soon as anything is digitally innovative, [data security] always comes right to my mind.

D2; no professional experience with VR

For one respondent with VR experience, data security played a rather subordinate role. One participant reasoned the following:

But when I say I always turn on the [VR] device and it does not store any data. I do not have to enter a name...just button up and go. Then I do not think that is problematic [in terms of privacy].

PT3; professional experience with VR

Furthermore, one participant said that “nowadays...data privacy must be accepted everywhere. That should be the lesser inconvenience” (MTF1; no professional experience with VR).

##### Complexity

Complexity as a characteristic of the innovation was found in a total of 71 text passages in the interviews, divided into the *complexity of VR hardware*, with 22 (31%) coded text segments, and the *complexity of VR software*, with 49 (69%) coded text segments. Health care professionals with or without VR experience did not differ in terms of the mere frequency of mentioning this criterion. However, individuals with VR experience were more likely to assess what may be disruptive about the hardware (eg, excessively heavy goggles). Participants described that VR technology should be user-friendly, intuitive, and robust against user error (OT1; professional experience with VR). VR technologies should be designed to be simple enough “...that if you are not that computer literate, you can get on with it quickly...” (LT1; no professional experience with VR). One interviewee stated that too much complexity in the system “can lead to failure [of VR technologies]” (PT2; no professional experience with VR). Furthermore, interview participants indicated that lack of functionality and applicability impact usability the most. For example, one participant explained the following:

My biggest concern is the applicability, whether this concept works well or still seems very, very beginning...[then it may be] I bought it [VR technologies] but will never use it.

PT3; professional experience with VR

In addition to the hardware quality, respondents ranked the software quality of VR technologies as important for potential adoption (PT2; no professional experience with VR). The VR technologies should be “transportable” (OT2 [professional experience with VR] and LT1 [no professional experience with VR]), “light” (OT2; professional experience with VR), “comfortable” (P1; professional experience with VR), and “robust” (ST1; no professional experience with VR). Those interviewees who were already VR users criticized that the VR glasses were “too heavy” (P1 and ST2; professional experience with VR), “the cables...[can] be a difficulty” (P1; professional experience with VR), and some glasses “hurt on the nose” (P1; professional experience with VR). Furthermore, one participant found the use of VR glasses uncomfortable when the “field of view [is] still very limited...and partly a bit blurry” (P1; professional experience with VR).

##### Trialability

Trialability refers to the degree to which an innovation can be tested or experimented with before full adoption. The trialability of technologies played a minor role in the interviews. It was mentioned a total of 12 times. Generally, individuals without VR experience expressed a desire to trial the technology beforehand. One interview participant expressed the following desire:

It would be good to test it [VR technology] beforehand. So that you know what you’re dealing with...So do I really want to use it?

D4; no professional experience with VR

##### Observability

By observability, we mean the proof of the results through evidence-based studies and the measurability of the results of VR technologies. Observability was found in a total of 35 coded text passages in the interviews, divided into the *lack of evidence-based studies*, with 15 (43%) coded text segments, and the *measurability of results with VR technologies***,** with 20 (57%) coded text segments. Respondents who did not use VR technologies professionally primarily cited a lack of studies on their evidence base (D2 and LT1; no professional experience with VR). One participant stated that “they [VR technologies] are not researched or proven with studies at all how much this does” (LT1; no professional experience with VR). In contrast, the interviewees who used VR technologies commented that “results can be seen directly” (PT7; professional experience with VR) in that “[the patients’] progress can be better displayed, and thus there is always a direct comparative control” (OT1; professional experience with VR). One participant added the following:

...range of motion, mobility...you can measure it very well.

PT7; professional experience with VR

Individuals with VR experience emphasized the significant advantage of measurable and comparable outcomes facilitated by VR. Conversely, individuals without VR experience expressed a desire for precisely these measurable outcomes.

##### Compatibility

Compatibility was found in a total of 58 coded text passages in the interviews. We understand compatibility ambiguously. On the one hand, compatibility is the extent to which VR technologies match the *existing values and needs of the health care professional*, with 84% (49/58) of the coded text segments. On the other hand, compatibility means the *interoperability and connectivity between technologies*, with 16% (9/58) of the coded text segments. Concerning the former definition, the most common concern was about changed therapist-patient relationships due to VR technologies. In total, 2 interviewees expressed the wish that therapy should continue to be “person to person...so that the social component is not missing” (MTF1 [no professional experience with VR] and ST2 [professional experience with VR]). Individuals without VR experience feared being replaced. In addition to compatibility with existing values and norms, interview participants cited concerns about poor interoperability of the VR system and other equipment at the facility (PT2 [no professional experience with VR] and D3 [professional experience with VR]). One physiotherapist expressed the following:

Maybe it also fails because of [lack of] connectivity.

PT2; no professional experience with VR

He expressed the desire that VR technologies “[should] be compatible with other devices.” Another interviewee added the following:

And then maybe it would be great if the VR medicine platform synchronizes as much as possible and does not have twelve operating systems.

D3; professional experience with VR

## Discussion

### Principal Findings

#### Overview

**Figure 2:**
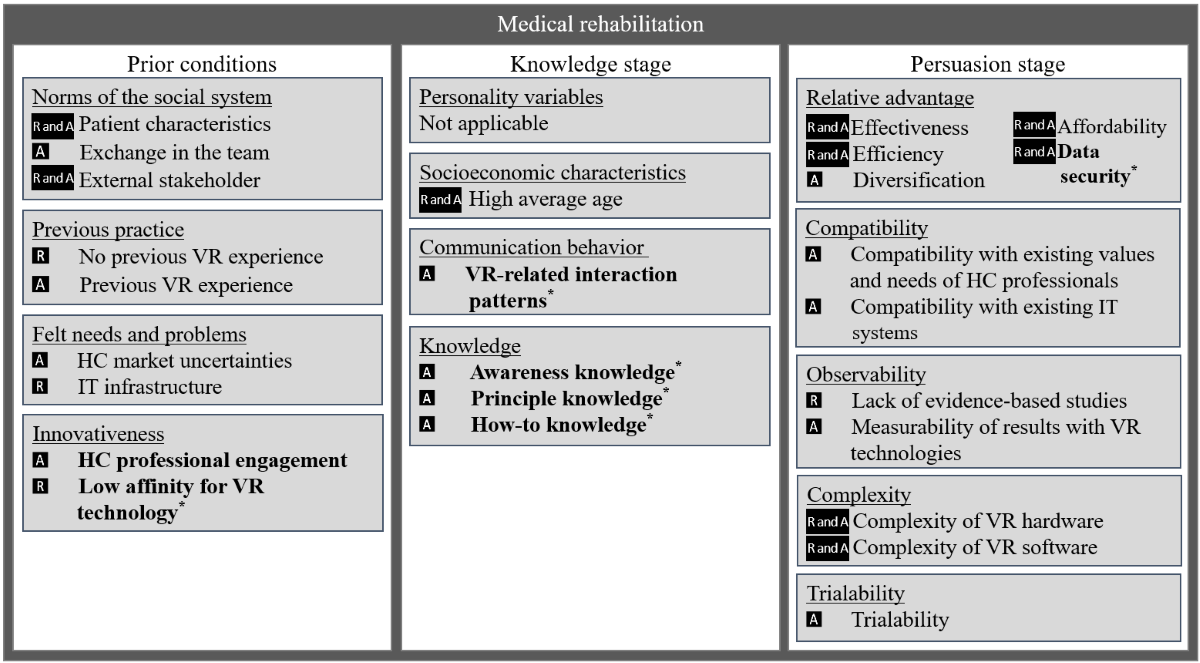
Contributing factors to resistance to and adoption of virtual reality (VR) technologies in medical rehabilitation. *Variations between VR-experienced innovators and nonexperienced laggards. A: adoption; HC: health care; R: resistance.

This study identified 26 factors that determine health care professionals’ adoption or nonadoption of VR technologies in medical rehabilitation (Figure 2). To achieve this goal, this study examined the attitudes of health care professionals with and without VR experience to gain a nuanced understanding of the diffusion of VR technologies in medical rehabilitation. This, in turn, aimed to enhance the overall understanding of the slow diffusion of innovations in health care. To this end, we followed the adopter categorization by Rogers [25], classifying the health care professionals into VR-experienced innovators and nonexperienced laggards.

First, we will discuss the characteristics of both groups. We will then illustrate how these insights necessitate contextualizing the adoption decision process, as Rogers [25] outlined, of VR technologies in medical rehabilitation. This involves considering the similarities and differences between the 2 adopter groups to improve the overall understanding of innovation dissemination in health care (see Figure 2, headings in bold). An overview of the key variations between VR-experienced innovators and nonexperienced laggards can be found in Multimedia Appendix 1.

#### Characteristics of VR-Experienced Innovators and Nonexperienced Laggards

We found evidence in our data supporting the characteristics of the generalized adopter categories by Rogers [25] in the diffusion of innovation theory. Health care professionals with VR experience exhibited a notable openness to VR technology and a great ability to deal with uncertainty related to this relatively new therapeutical option.

Participants with VR experience tried to develop strategies for potential problems (eg, technical failure) and, thus, expressed solution-oriented thoughts on VR implementation, such as training courses to deal with new technologies or the dissemination of knowledge through more educational work in targeted marketing campaigns. Such a solution-focused stance is typical of innovators as conceptualized in the diffusion of innovation theory, demonstrated in a study by Haun et al [55] in the health care sector on the adoption of video consultations by general practitioners. Another characteristic that, according to the diffusion of innovation theory, is related to the ability to innovate is the opinion leadership of innovators. One therapist, for example, reported using VR technologies at school to raise awareness.

Health care professionals without VR experiences in our sample anticipated several problems (eg, concerns regarding familiarization time and training effort due to the introduction of new technologies). They did not mention any possible solutions to their concerns. While non–VR users expressed criticism toward the outdated curriculum and therapy methods, their inclination toward embracing change appeared limited. The interviews elucidated that participants without VR experience tended to conform to existing practices rather than engage in forward-thinking innovation, indicating an indirect demand that managers set an example in the use of technologies such as VR. Respondents without VR experience even expected knowledge transfer of technologies such as VR by managers and rehabilitation organizations (top-down push).

The results revealed more than just differences between the 2 adopter groups. Surprisingly, the respondents agreed on the factors that promote the adoption of VR technologies. For example, both groups cited the efficient use of technology or the measurability of therapy outcomes as promoting factors. Respondents without VR experience often highlighted the same factors that respondents with VR experience mentioned as major advantages when using the technology. This makes it clear that the advantages of VR technologies over conventional therapy methods in medical rehabilitation are not just utopian dreams but are being realized in practice.

These findings suggest that, in implementation and dissemination efforts, health care professionals’ concerns over perceived negative aspects of VR (eg, cost and training effort) need to be addressed, whereas specifically promoting positive aspects (eg, being able to work faster and time efficiency) may be of lesser importance as these are likely self-apparent to practicing health care professionals.

#### Limited Level of Awareness Knowledge and Fear of the New

In the health care sector, innovation diffusion unfolds at a fundamentally slower pace than in other sectors [56]. Health care professionals are more deeply engaged in their clinical work than leaders in other sectors [57], thus having less time to familiarize themselves with digital innovations, impacting their readiness for adoption.

Research findings indicate that a limited level of knowledge among health care professionals influences the adoption of these technologies [17,19,58]. Our results regarding the readiness of health care professionals to adopt VR technologies reveal differences between individuals who had gained experience with VR technologies and those who had not. In particular, the characteristics of the innovation, which significantly influence adoption behavior [25], were differently evaluated by the two groups. For instance, individuals without VR experience perceived the costs and the effort of training as high and considered them as obstacles to their use. Conversely, individuals with VR experience considered the costs and the training effort affordable and were not deterred by them. As VR is no longer a new technology, the acquisition costs have fallen [59]. This could lead to the assumption that health care professionals without experience cannot estimate the costs, rely on incorrect or outdated knowledge, and abandon the effort associated with introducing new technologies.

Furthermore, non–VR users expressed concerns about being replaced by VR technologies or the physician-patient relationship possible being negatively affected. This phenomenon is termed *technophobia* [60], encompassing any hesitation, reservation, skepticism, concern, fear, or dread regarding implementing technology in clinical practice [61]. These concerns were irrelevant among individuals who had gained experience with VR technologies. Studies indicate that the frequency of use increases comfort with the technology and willingness to use it [62]. Ultimately, a lack of knowledge exacerbates concerns and, thus, can hinder potential use.

Both innovators and laggards expressed the need for evidence-based studies to reduce uncertainties regarding the efficacy and safety of VR technologies. This is an interesting finding as, according to Rogers [25], information acquisition in the adoption decision process often lacks systematic reliance on scientific insights. This less science-oriented approach is also evident in medical practice, where health care professionals increasingly incorporate individual assessments into the selection and implementation of digital innovations rather than accessing evidence-based insights [56]. This could be attributed to challenges in researching and evaluating information on digital innovations. The question arises of how, considering the time constraints of health care professionals, the efficacy and safety of VR technologies can be effectively communicated to provide them with easy access to evidence-based information sources.

In this study, knowledge transfer and communication between the two adopter groups appeared to be difficult, in particular as respondents from the group of VR-experienced health care professionals saw themselves as opinion leaders but not as responsible for passing on knowledge. The results show a lack of adoption groups in between the innovators and laggards, such as the early majority or late majority, who contribute to the dissemination of knowledge. These two groups are typically well connected with their colleagues and could mitigate this lack of communication and knowledge transfer. According to Rogers [25], they serve as a bridge between the adopter groups and, consequently, contribute to the dissemination of technologies such as VR. Overall, adopter group–specific and balanced information dissemination about VR technologies is necessary to promote adequate use in health care and address potential concerns.

#### Data Security

Data security is already well recognized and researched in the literature on technology adoption in health care [63]. However, the rise of VR technologies introduces new and amplified concerns, particularly regarding the storage and use of sensitive patient data [22].

The interview findings reveal a discrepancy in the perceived importance of data security between participants with and without VR experience. Those with VR experience tended to assign less significance to data security concerns than their counterparts lacking VR experience, who expressed heightened apprehension. In most cases, the reason given was that “medical data is stored [through the use of VR technologies]” (MTF1; no professional experience with VR). However, using VR technologies does not always involve collecting and storing sensitive patient data. This was also argued by one participant, who said the following:

...I always switch on the [VR] device, and it does not store any data. I do not have to enter a name...just press the button and go.

PT3; professional experience with VR

Therefore, it can be concluded that medical staff without VR experience lack knowledge about VR and data protection and subconsciously use this argument to avoid using VR.

Studies have shown that health care professionals lack awareness of data security measures [64]. This lack of knowledge typically leads to an absence of safety risk assessments, meaning that data protection issues are not adequately considered in practice. As a result, many health care professionals do not exhibit a strong sense of caution or concern regarding data security in their day-to-day work [65].

### Theoretical Contribution and Practical Implications

Our contribution to theory is 2-fold. First, this study advances the diffusion of innovation theory literature and follows the call by Burton-Jones and Volkoff [66] for context-specific rather than general perspectives. We found evidence for the applicability of the adoption decision process by Rogers [25] to the context of medical rehabilitation by identifying 26 factors that influence the adoption or rejection by health care professionals of VR technologies in medical rehabilitation. Therefore, our study provides valuable findings that can be used to promote VR technology diffusion in the health care sector. Second, our study contributes to theory by going beyond a hypothetical use scenario by categorizing health care professionals into 2 user groups: those with professional experience and those without professional experience with VR technologies. In line with previous research [18,58], our study demonstrates that the inclusion of actual use experiences, as opposed to considering the intention to use, enhances the explanatory power of the findings and provides a deeper understanding of the barriers to and facilitators of VR adoption. This nuanced understanding deepens the diffusion of innovation theory by emphasizing the importance of adapting adoption strategies while accounting for user-specific factors such as knowledge gaps and individual characteristics. For example, while studies have identified perceived costs and training efforts as significant barriers [17,19,58], our results reveal that these concerns are primarily held by non–VR users and are less pronounced among those with VR experience. The 2 user groups did not agree on some points, which can be attributed to different levels of knowledge and individual characteristics leading to different perceptions and adoption barriers. These differences highlight the need for adopter-specific communication strategies to address unique concerns and foster the diffusion of VR technologies. Key recommendations for communication strategies include empowering innovators to take on leadership roles, share experiences through peer networks, and drive solution-oriented approaches for VR adoption. For laggards, the focus should be on education and training, addressing concerns about benefits, costs, and data security and alleviating technophobia by emphasizing VR as a tool to enhance, not replace, human roles in patient care. Structured mentorship and training programs can facilitate knowledge transfer from innovators, helping bridge the gap.

In addition to the theoretical contributions, we derived 2 practical implications. First, our results suggest that health care professionals desire enhanced education through various channels, including from leaders, as well as during medical education and through events and social media. A stronger focus on VR technologies in medical rehabilitation by politicians, health insurers, and health care organizations can contribute to the dissemination of knowledge. Through these stakeholders, education and knowledge can be offered to reduce fear and answer questions about VR technologies. This can be supported by disseminating evidence-based information via rehabilitation-specific journals and the involvement of associations related to the therapy profession to overcome the knowledge gap between health care professionals with and without experience. By anchoring VR technologies in the German S3 guidelines, the decision-making process of health care professionals regarding a suitable VR technology can be supported. Second, given the heightened data security concerns among non–VR users, providing clear, accurate information on data protection measures associated with VR technologies is essential. For instance, Microsoft HoloLens offers a comprehensive list of security protocols, including the use of BitLocker for encrypting user drives or a Trusted Platform Module for enhancing security through encryption and decryption [67]. These measures, along with others, should be considered and communicated effectively to health care professionals. There is a clear need for future educational initiatives aimed at informing health care professionals about the specific technologies that require strict attention to data security. Developing guidelines and protocols that ensure data security can help alleviate fears and misconceptions about VR use.

### Limitations

This study has several limitations that suggest potential areas for future research. The first limitation results from the qualitative research approach, which prevents us from drawing conclusions about entire populations and, thus, affects our results’ generalizability. To mitigate the limitation of a small sample size, we selected a diverse sample in terms of age, sex, and profession. By leveraging the strengths of the qualitative approach, we were able to capture rich and diverse data in a new area of research that quantitative studies may not capture. Thereby, we followed the call for research by Halbig et al [13], who recommend interviews to obtain more in-depth information on VR technology adoption or nonadoption by health care professionals. Second, regarding the diffusion of innovation theory, our results also provide a basis for further research. The respondents assessed the effect of the identified factors (whether they were resistant to or acceptant of them) very differently. A quantitative study with a large number of participants could investigate which factors are more likely to lead to adoption or resistance. In addition, a survey of individual personality traits using established scales such as the *Big Five* could allow for a clearer distinction to be made among the 5 adopter groups by Rogers [25]. The third constraint of our study stems from the data analysis approach of the QCA, which comes with the potential bias of the researcher. Personal bias can occur while coding, categorizing, and interpreting textual content. We mitigated this bias by engaging in regular sessions with the author team to discuss memos and codes.

### Conclusions and Further Research

This study explored the factors influencing health care professionals’ adoption or nonadoption of VR technologies in medical rehabilitation based on their previous experiences. While VR has shown potential benefits in enhancing patient care and rehabilitation outcomes, its clinical adoption remains limited, primarily due to concerns regarding data security or perceived cost, which may stem from insufficient knowledge among potential users. By analyzing 23 semistructured interviews with health care professionals in Germany, we identified 26 factors within the 4 categories by Rogers [25] that contribute to the adoption or nonadoption of VR technologies. By distinguishing between VR-experienced innovators and nonexperienced laggards, our findings emphasize the importance of addressing these groups’ unique needs to promote wider adoption. VR-experienced professionals tended to exhibit characteristics such as openness to new technologies, solution-oriented thinking, and thought leadership, whereas non–VR users were more focused on barriers and relied on top-down knowledge transfer. Nevertheless, both groups agreed on the factors that promote the adoption of VR technologies, such as the efficient use of technology and the measurability of therapy outcomes. This research contributes to the diffusion of innovation theory by identifying important differences between VR-experienced and nonexperienced health care professionals. These insights can support the development of targeted communication strategies to promote VR adoption in health care. Further research is needed to develop and test specific communication approaches based on these findings. Moreover, longitudinal studies could examine how knowledge of and attitudes toward VR evolve over time and assess the long-term impact of targeted training programs and communication strategies on the adoption rate. Expanding investigations to diverse health care settings and populations, as well as exploring organizational dynamics such as team structures and leadership support, could provide a broader and more comprehensive understanding of the factors influencing VR adoption. In addition, examining diverging factors such as a high average age and their influence on technology adoption could offer valuable insights to guide the effective implementation of new technologies.

## Appendix

Multimedia Appendix 1 Overview of key variations between virtual reality–experienced innovators and nonexperienced laggards.

## Abbreviations

QCA: qualitative content analysis
VR: virtual reality
