# Supplementary material for: The Influence of Previous Experience on Virtual Reality Adoption in Medical Rehabilitation and Overcoming Knowledge Gaps Among Health Care Professionals: Qualitative Interview Study
Source: J Med Internet Res. 2025 Apr 30;27:e62649. doi: 10.2196/62649 (PMC12079057; doi:10.2196/62649)
Supplement: Multimedia Appendix 1 [file jmir_v27i1e62649_app1.docx]

**Multimedia Appendix 1. Overview of Key Variations between VR-Experienced "Innovators" and Nonexperienced "Laggards".**

| **Rogers’ Category** | **Theme** | **Innovators (VR-Experienced)** | **Laggards (Non-VR-Experienced)** | **Explanation** |
| --- | --- | --- | --- | --- |
| **Health care professionals characteristics** | **Openness to Technology** | High openness; proactive in adopting new technology; dealing with uncertainty | Limited openness; more resistant in adopting new technology | Innovators are solution-oriented and proactive, whereas laggards resist change due to lack of familiarity and knowledge. |
|  | **Problem-Solving Approach** | Focus on addressing challenges of VR adoption (e.g., technical failures, training) | No mention of solutions for successful VR adoption; resistant to change due to perceived challenges | Innovators focus on developing strategies for overcoming potential issues, while laggards see the challenges as obstacles without suggesting solutions. |
|  | **Knowledge Acquisition** | High knowledge; view VR as feasible | Low knowledge; perceive VR as complex to implement | Innovators have direct experience and are comfortable with VR's training efforts, while laggards lack sufficient information, which exacerbates concerns. |
| **Characteristics of VR technologies** | **Training and Familiarization** | Willing to engage in training and education to enhance knowledge | Concerned about the time and effort required for training | Innovators see training (and time) as an opportunity, whereas laggards view it as an obstacle. |
|  | **Perception of Costs** | See costs as reasonable; lower perceived financial risk | See costs as high; perceive financial risk | Innovators have direct experience with VR technologies and are more willing to accept the costs. Laggards tend to rely on outdated information, increasing their concern about costs. |
|  | **Technological Displacement** | Little to no concern about being replaced by technology | Fear of job displacement or reduced human interaction | Laggards express technophobia, fearing that technology may replace human involvement, while innovators do not share this concern. |
|  | **Evidence-Based Studies** | Support the need for evidence to reduce uncertainty | Also desire evidence but lack access to or knowledge of research | Both groups acknowledge the need for evidence but differ in their approach to acquiring it. Laggards tend to rely on anecdotal evidence and experience, while innovators seek scientific validation. |
|  | **Data Security Concerns** | Less concerned about data security; familiarity with VR's data handling | High concern about the security of medical data | Innovators understand how VR works and its data handling capabilities, while laggards have misconceptions or lack awareness about data security in VR technologies. |
| **Communication**  **strategies** | **Communication and Knowledge Transfer** | See themselves as opinion leaders but not responsible for spreading knowledge | Seek knowledge transfer through management or external sources | There is a lack of communication between innovators and laggards. Innovators are less inclined to spread their knowledge, while laggards rely on top-down communication for guidance. |
